# miR-1275 targets MDK/AKT signaling to inhibit breast cancer chemoresistance by lessening the properties of cancer stem cells

**DOI:** 10.7150/ijbs.74227

**Published:** 2023-01-01

**Authors:** Xu Han, Minghui Li, Jin Xu, Jingyue Fu, Xinyang Wang, Jingyi Wang, Tiansong Xia, Shui Wang, Ge Ma

**Affiliations:** 1 Department of Breast Surgery, The First Affiliated Hospital with Nanjing Medical University, 300 Guangzhou Road, 210029, Nanjing, China.; 2 Jiangsu Key Lab of Cancer Biomarkers, Prevention and Treatment, Jiangsu Collaborative Innovation Center for Cancer Personalized Medicine, School of Public Health, Nanjing Medical University, 211166, Nanjing, China.; 3 Department of Breast and Thyroid Surgery, Nanjing First Hospital, Nanjing Medical University, 210029 Nanjing, China.; 4 Department of Thyroid and Breast, The Second Affiliated Hospital of Nantong University, 226000, Nantong, China.; 5 Department of Breast Surgery, The Affiliated Changzhou No. 2 People's Hospital of Nanjing Medical University, 29 Xinglong Lane, 213003, Changzhou, China.

**Keywords:** liquid biopsy, chemoresistance, miR-1275, CRISPR/Cas9, cancer stem cell

## Abstract

Chemoresistance is a major obstacle in the neoadjuvant chemotherapy (NCT) of locally advanced breast cancer (LABC). Identification of miRNAs as prognostic biomarkers may help overcome chemoresistance of breast cancer (BC). This study aimed to evaluate the expression level of miR-1275 in plasma samples and its biological functions in the chemoresistance of BC. The expression levels of miR-1275 in plasma samples and cells were measured by RT-qPCR. CRISPR/Cas9-mediated gene editing was used to construct miR-1275 knock-out cells in MCF-7. We found that miR-1275 was significantly downregulated in plasma from patients resistant to chemotherapy and in chemoresistant BC cell lines, while patients with low levels of miR-1275 showed poor overall survival. miR-1275 knock-out promoted chemoresistance in BC cells by increasing the properties of cancer stem cells (CSCs). Mechanistically, we identified that MDK was determined to be direct downstream protein of miR-1275 which initiated PI3K/Akt signaling in breast cancer cells. We demonstrated that the high expression level of miR-1275 in plasma predicted better response to NCT. The reduction of miR-1275 promoted BC cells chemoresistance by increasing CSCs properties via targeting MDK/AKT axis. The potential of miR-1275 as a new prognostic biomarker and therapeutic target of BC patients was identified.

## Introduction

Breast cancer (BC) is a commonly diagnosed type of cancer worldwide, and the leading cause of cancer-related deaths among women 1. Multidisciplinary treatment for locally advanced breast cancer (LABC) patients includes preoperative neoadjuvant chemotherapy (NCT), surgery, and adjuvant systemic and local treatment 2. As a first-line treatment for LABC, NCT is increasingly preferred in early cases 3. The advantages of NCT include enabling breast conserving surgery by shrinking the breast lesion, eliminating micrometastasis, evaluating drug resistance, and estimating the prognosis 4. In spite of this, more than 80% of patients who receive NCT do not achieve a pathological complete response 5. Therefore, there is concern that NCT may delay definitive treatment for operable LABC patients, thus leading to drug resistance and systemic metastases in the future. Consequently, dynamic monitoring treatment response at an early stage and therapeutic intervention of chemotherapy resistance will increase the effectiveness of NCT.

Currently, liquid biopsies as a source of biomarkers have raised people`s interest, as they offer a minimally invasive method which costs less and minimizes the invasiveness of tissue sampling 6, 7. In the previous, our research group established an efficient evaluation model to evaluate the prognostic role of circulating tumor cells (CTCs) in the response to NCT, and we used the same batch of samples in this study 8. MicroRNAs (miRNAs) are small (~22 nucleotides), single stranded, non-coding RNA molecules. Circulating microRNAs exist in body fluids as free, unbound miRNAs, miRNAs bound to proteins, or membrane-wrapped miRNAs such as in microvesicles and exosomes 9. Their characteristics of stability, ease of detection, and minimal invasiveness solidify their utility as promising diagnostic, prognostic, and predictive biomarkers for BC 10.

A variety of mechanisms are involved in chemoresistance, including alteration of non-coding RNAs, drug export transporters, DNA repair mechanisms, cancer stem cells, resistance to apoptosis, self-sufficiency for growth factor signaling, angiogenic switch, and immunological pathways 11. As a result of post-transcriptional regulation, miRNAs can alter the expression of multiple mRNAs, including oncogenes and tumor suppressor genes 12. It has been documented that microRNAs have a fundamental role in several physiological and pathophysiological cellular processes, such as proliferation, differentiation, apoptosis, autophagy, migration, and chemoresistance 13. Aside from that, cancer stem cells (CSCs) encompass a small group of cells that remain dominant during rapid tumor cell growth, which evades chemical compounds, thus leading to tumor recurrence or chemoresistance 14. Consequently, understanding the molecular mechanisms which lead to chemoresistance in NCT is essential to develop efficiency of BC treatments.

In this study, we enrolled 30 LABC patients with similar tumor stages that accepted the same NCT based on anthracycline and taxane drugs. We dynamically monitored the expression levels changes of miRNAs in peripheral blood before and after treatment, and found that the expression level of miR-1275 in NCT-resistant patients was significantly lower than that of NCT-sensitive patients before NCT. We investigated the biological functions of miR-1275 in LABC chemoresistance *in in vitro* and *in vivo*, and further identified Midkine (MDK) as a direct target gene of miR-1275. Our results confirmed for the first time that miR-1275 inhibited epirubicin resistance via regulation of the miR-1275/MDK/AKT axis to lessen the properties of cancer stem cells, indicating that miR-1275 has potential to be a suppressor in BC chemoresistance.

## Results

We enrolled 30 LABC patients with similar tumor stages that received the same NCT treatment with epirubicin, cyclo-phosphamide and docetaxel (epirubicin 90 mg/m^2^ iv D1, cyclophosphamide 600 mg/m2 iv D1 on a 21-day cycle for four cycles, then docetaxel 80 mg/m2 iv D1, on a 21-day cycle for four cycles). In the light of the Miller-Payne system, 15/30 patients achieved <30% loss of tumor cells (3/30 grade 1 and 12/30 grade 2), and the remaining 15 patients were classified as grade 3-5 (14, 0, and 1 as grades 3, 4, and 5, respectively). On the basis of the Miller-Payne system, the NCT response were independent of age, HER-2 status, molecular subtype, or lymph node metastasis in this study (Table 1).

**Figure 1:**
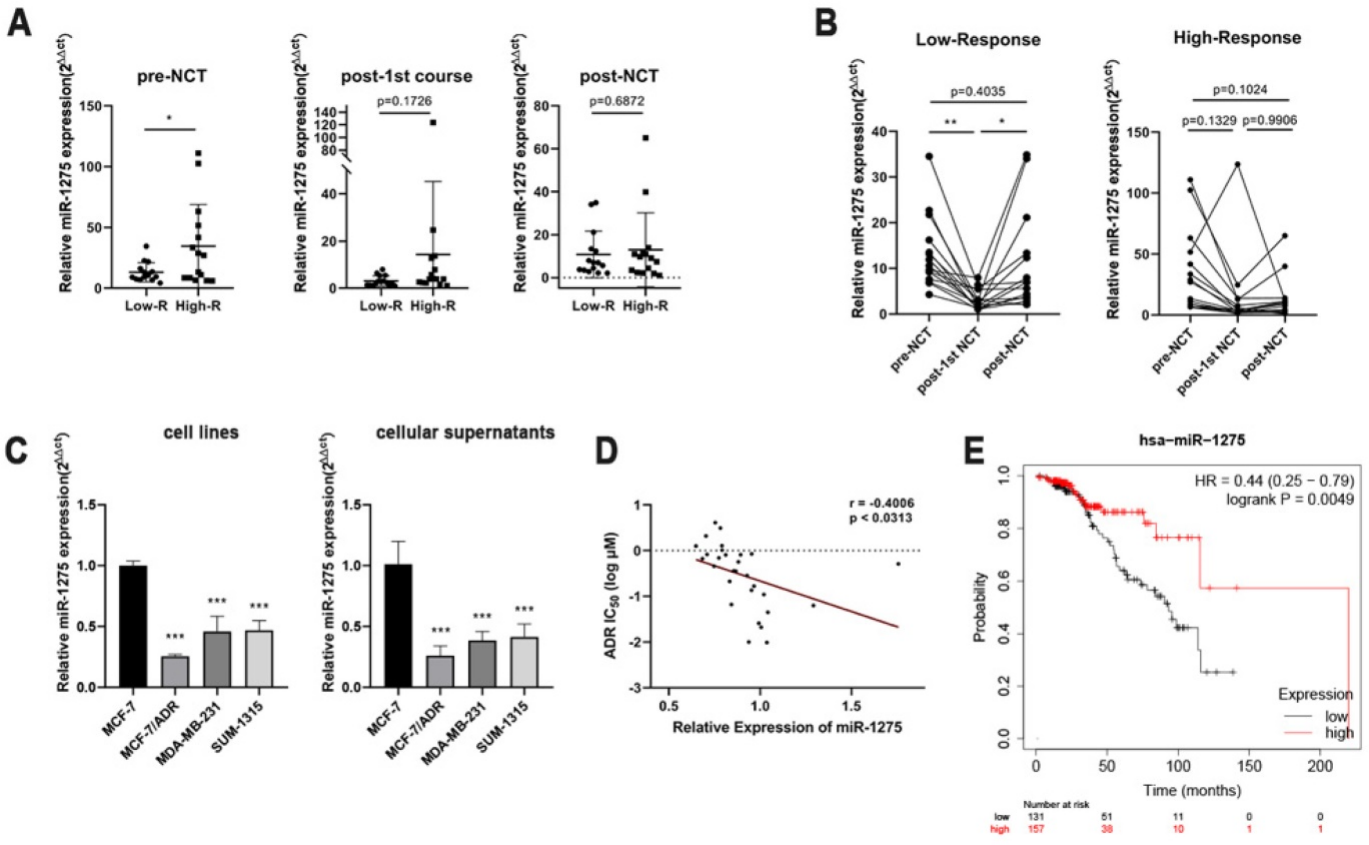
** miR-1275 was significantly downregulated in chemoresistant BC patients and acted as a prognostic factor. A.** Comparison of expression levels of miR-1275 in different time points during NCT between the Low-R group (Miller-Payne grades 1 and 2) and the High-R group (Miller-Payne grades 3, 4 and 5). **B.** Comparison of expression levels of miR-1275 in different response groups during NCT. **C.** The expression levels of miR-1275 in BC cell lines and cellular supernatants. **D.** The correlation between miR-1275 expression and the IC50 value of epirubicin in 29 breast cancer cell lines in the CCLE and GDSC databases. **E.** Data of miR-1275 was obtained from the TCGA database. Lower expression of miR-1275 was correlated with a worse overall survival in patients with LABC. *p < 0.05, **p<0.01, ***p < 0.001. The data expressed as the mean ± SD.

We dynamically monitored a series of expression level changes of miRNAs in peripheral blood before and after treatment, and we were attracted by miR-1275. Prior to the start of NCT, the expression level of miR-1275 were significantly higher in High-R group than that in the Low-R group (Fig. 1a). During NCT, a significant decrease in the Low-R group was observed after the first NCT course compared with the miR-1275 level before NCT (Fig. 1b). We also noticed that in the Low-R group compared with the expression level after first cycle, miR-1275 significantly increased after 8 cycles of chemotherapy, which was still lower than that before chemotherapy (Fig. 1b). At the same time, no significant difference in High-R group was found at the three time points during NCT (Fig. 1b).

Due to the difficulty of obtaining tissue samples from patients undergoing NCT, we detected the expression levels of miR-1275 in different BC cell lines with different malignant behaviors by RT-qPCR, to validate the association between the expression pattern of miR-1275 and BC chemoresistance. At the same time, expression levels of miR-1275 in the supernatants of BC cell lines were analyzed by RT-qPCR. As expected, the epirubicin-resistant BC cell (MCF-7/ADR) had reduced miR-1275 expression compared with the parent BC cell (MCF-7), and miR-1275 exhibited a lower expression level in the other two TNBC cells (SUM-1315 and MDA-MB-231) than in MCF-7 cells (Fig. 1c). The results of the supernatants of BC cell lines were consistent with the expression levels in BC cells (Fig. 1c). To verify the correlation of the expression level of miR-1275 and the epirubicin sensitivity of breast cancer cells, we observed the expression levels of miR-1275 in various breast cancer cells from the CCLE database and then searched the IC50 values of epirubicin in these breast cancer cells from the GDSC database. The results showed that the expression level of miR-1275 was negatively correlated with the epirubicin resistance of breast cancer cells (p <0.0313, r = -0.4006) (Fig. 1d). The overall survival of 288 patients with LABC about miR-1275 was obtained from the TCGA database and analyzed with Kaplan-Meier Plotter. The high expression of miR-1275 was associated with a better overall survival (157 high level and 131 low level, p = 0.0049), and HR was 0.44 (95% CI 0.25-0.79) (Fig. 1e).

Subsequently, we attempted to examine the effects of miR-1275 on BC chemoresistance. To do so, we successfully constructed miR-1275 overexpression MCF-7/ADR, SUM-1315 and MDA-MB-231 cells with miR-1275 mimics lentivirus, which were confirmed by RT-qPCR (Supplementary Fig. 1a). At the same time, we used CRISPR/Cas9 to construct miR-1275 knock-out MCF-7 cells (Supplementary Fig. 1b). The sequence character of miR-1275 was confirmed in MCF-7 cells with Sanger sequencing carrying gene deletion at the gRNA-targeting region (Supplementary Fig. 1c). In addition, RT-qPCR confirmed no expression of miR-1275 in gene-edited MCF-7 cells as well (Supplementary Fig. 1d).

**Figure 2:**
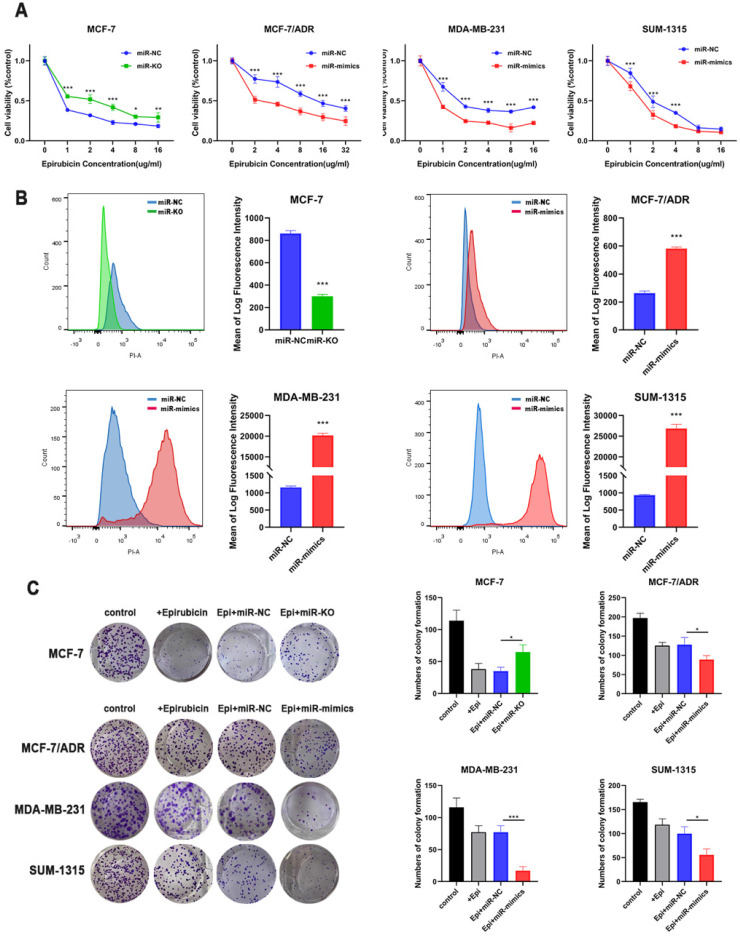
** miR-1275 overexpression inhibited chemoresistance *in in vitro*. A.** Drug resistance was examined by CCK-8 assays in miR-1275 knocked-out cells or cells transfected with miR-1275 mimics. **B.** The fluorescence intensity of epirubicin was detected by flow cytometry, showing intracellular epirubicin accumulation. **C.** The cells were treated with epirubicin, and colonies were stained with crystal violet. *p < 0.05, **p<0.01, ***p < 0.001. The data expressed as the mean ± SD.

CCK-8 cytotoxicity test was used to detect changes in the drug sensitivity. After overexpressing miR-1275, cells revealed an increase in chemosensitivity. However, after miR-1275 was knocked out, the cells' drug sensitivity was significantly decreased (Fig. 2a). Next, we used flow cytometry to examine drug accumulation in cells. The overexpression of miR-1275 significantly increased the accumulation of epirubicin in cells, while silencing of miR-1275 cut down the accumulation of epirubicin in cells (Fig. 2b). In addition, colony formation assays were used to evaluate the role of miR-1275 on BC cell epirubicin resistance. The number of BC cell colonies was significantly decreased in miR-1275 overexpressed cells, however, increased in miR-1275 knocked-out cells (Fig. 2c). To sum up, these findings suggest that miR-1275 promotes epirubicin sensitivity in BC cells *in in vitro*.

**Figure 3:**
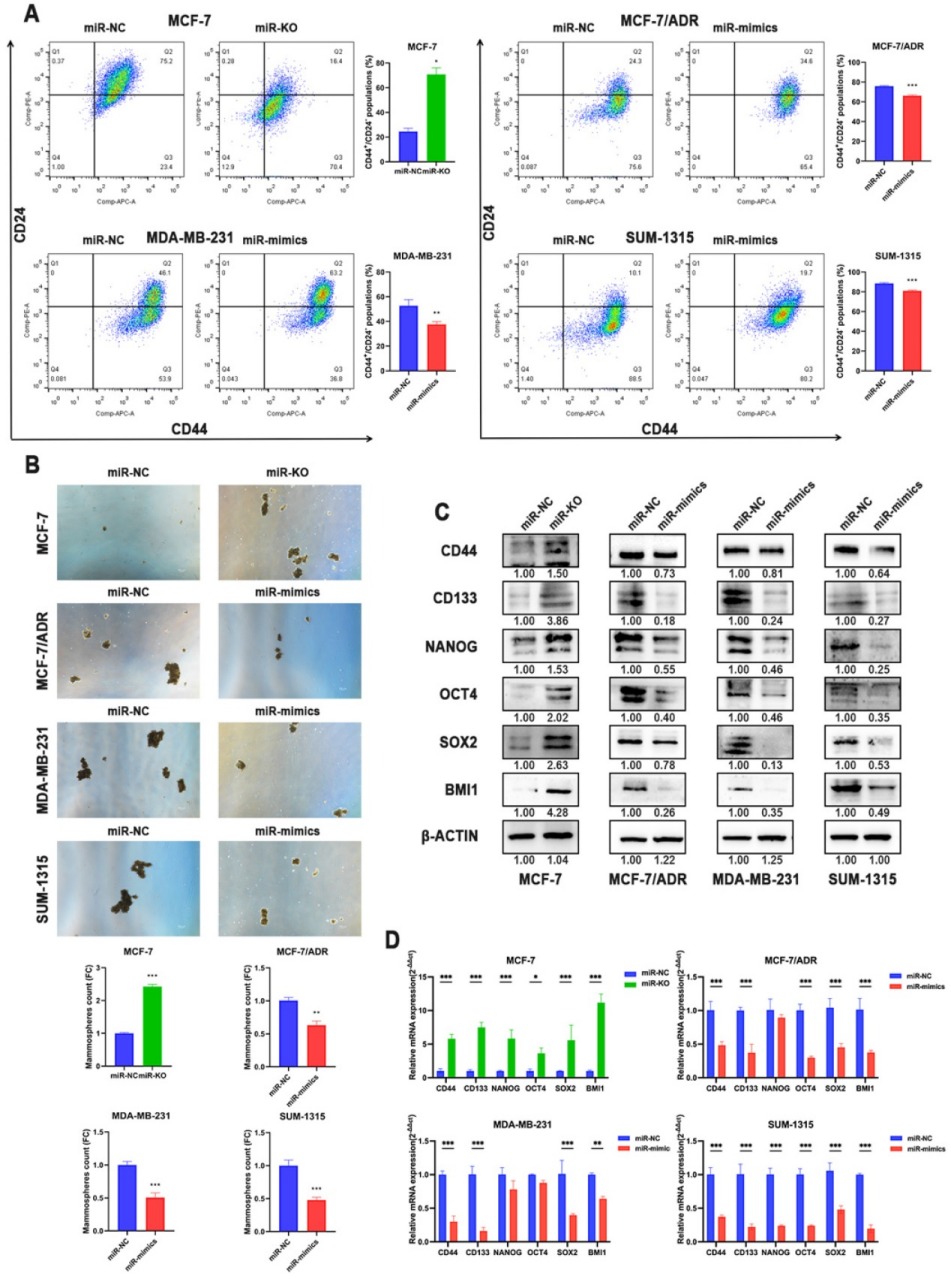
** Overexpression of miR-1275 reduced CSCs properties *in in vitro*. A.** The expression levels of CD44 and CD24 in cells after transfection were analyzed by flow cytometry analysis. Cells were stained with anti-CD44-APC and anti-CD24-PE. **B.** Spheroids formation assay was performed, and the representative pictures were shown. **C.** Protein expression of stem-related genes such as CD44, CD133, NANOG, OCT4, SOX2 and BMI1 in MCF-7, MCF-7/ADR, MDA-MB-231 and SUM-1315. **D.** mRNA expression of stem-related genes such as CD44, CD133, NANOG, OCT4, SOX2 and BMI1 in MCF-7, MCF-7/ADR, MDA-MB-231 and SUM-1315. *p < 0.05, **p<0.01, ***p < 0.001. The data expressed as the mean ± SD. Scale bar, 100 µm.

Multiple evidences suggested that CSCs play a critical role in the induction and maintenance of chemotherapy resistance in tumors, which is the cause of the failure of chemotherapy in tumor therapy, suggesting that these two cellular courses are closely related. Therefore, the effects of miR-1275 on CSCs characteristics in BC cells were further evaluated. Flow cytometry revealed that miR-1275 overexpression reduced the populations of CD44^+^/CD24^-^ in BC cells compared to negative control, while knocking-out miR-1275 increased the fraction of CSCs (Fig. 3a). Furthermore, spheroids formation assays were performed and the results showed that BC cells overexpressing miR-1275 possessed decreased spheroids formation ability, while silencing miR-1275 increased spheroids formation ability (Fig. 3b). The influence of miR-1275 levels on stemness-associated genes in BC was also investigated. The RT-qPCR and WB results proved that silencing miR-1275 reduced the expression of stemness-related genes such as CD44, CD133, Nanog, OCT4, SOX2 and BMI1 in BC cells, while upregulating miR-1275 reduced the expression of these stem cell factors (Fig. 3c-d). Taken together, our results indicated that miR-1275 overexpression undermines the CSC properties *in in vitro*.

**Figure 4:**
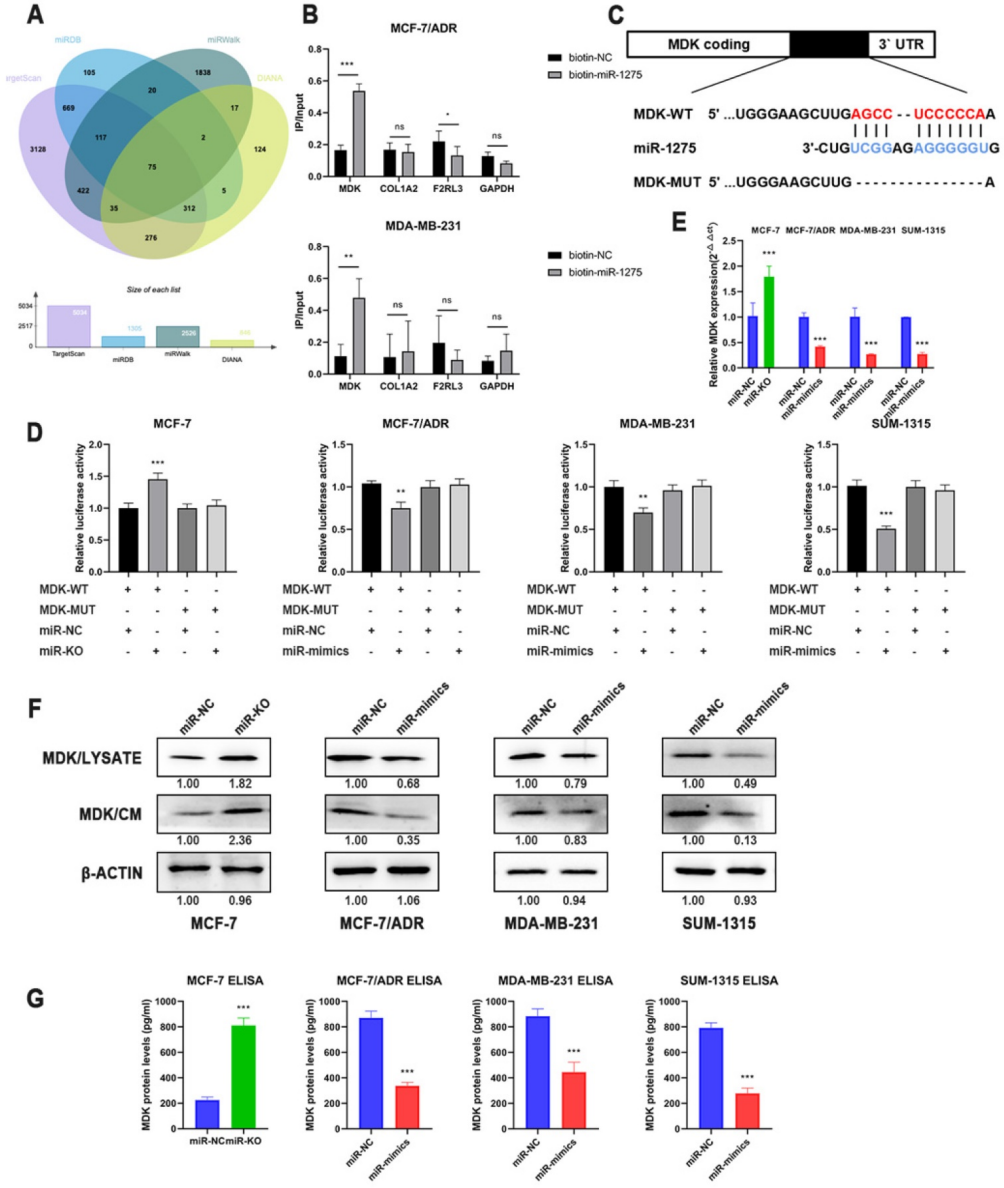
** MDK was a direct downstream target of miR-1275. A.** Venn diagram of predicted miR-1275 targets by 4 programs (TargetScan, miRDB, miRwalk and DIANA). **B.** The 3'-end biotin-labeled miR-1275 mimics and negative control were transfected into MCF-7/ADR and MDA-MB-231 cells, and the mRNA levels of MDK, COL1A2, F2RL3 and GAPDH were quantified by RT-qPCR, and the relative immunoprecipitate/input ratios were plotted. **C.** The luciferase reporter constructed that had either a MDK-WT or MDK-MUT sequence of the miR-1275 binding site. **D.** The luciferase reporter containing wild-type MDK-WT or MDK- MUT was transfected into BC cells correspondingly. Luciferase activity was determined by the dual luciferase assay. **E.** The mRNA level of MDK in BC cells after the transfection of miR-1275 were determined with RT-qPCR. **F.** The protein expression levels of MDK in cell lysate and conditioned media in BC cells after the transfection of miR-1275 were determined with western blot. **G.** The protein expression levels of MDK in conditioned media in BC cells after the transfection of miR-1275 were determined with ELISA assay. *p < 0.05, **p<0.01, ***p < 0.001. The data expressed as the mean ± SD.

To explore the latent mechanism of miR-1275, we searched the miRNA bioinformatics prediction websites (TargetScan, miRWalk, miRDB and DIANA) and detected these genes expression level in TCGA database, thus identified several feasible targets of miR-1275 (Fig. 4a). The 3'-end biotinylated miR-1275 mimics and negative control were transfected into MDA-MB-231 and MCF-7/ADR cells. The mRNA bound to miR-1275 was captured with streptavidin magnetic beads, and then the mRNA levels of COL1A2, MDK, F2RL3 and GAPDH were quantified by RT-qPCR. After plotting the relative immunoprecipitate/input ratios, MDK was recognized as a potential target (Fig. 4b). As secreted protein, MDK can function as both cytokine and growth factor, which adjusts many processes like cell survival, cell growth, tissue regeneration and cell differentiation. To determine whether MDK might be the downstream of miR-1275, luciferase reporter gene assay was performed. After obtaining possible binding sites from the database, luciferase reporter vectors MDK-WT and MDK-MUT containing miR-1275 binding site sequences were constructed (Fig. 4c). In MCF-7/ADR, SUM-1315 and MDA-MB-231 cells, the relative activity of luciferase was decreased after transfection of miR-1275 mimic and MDK-WT, while the relative activity of luciferase was not decreased after transfection of MDK-MUT (Fig. 4d). Meanwhile, knocking-out of miR-1275 significantly increased luciferase activity in MCF-7 cells (Fig. 4e). Luciferase reporter gene analysis showed that miR-1275 directly targeted the predicted sites of MDK mRNA. Furthermore, the data of RT-qPCR and Western blots revealed the downregulated MDK expression presented in overexpressed miR-1275 cells, and MDK levels were increased in miR-1275 knocked-out cells (Fig. 4e-f). As MDK is secreted protein, we detected the MDK protein levels in conditioned media using MDK ELISA assay and Western blot, which were similar to the result in the cells (Fig. 4f-g).

**Figure 5:**
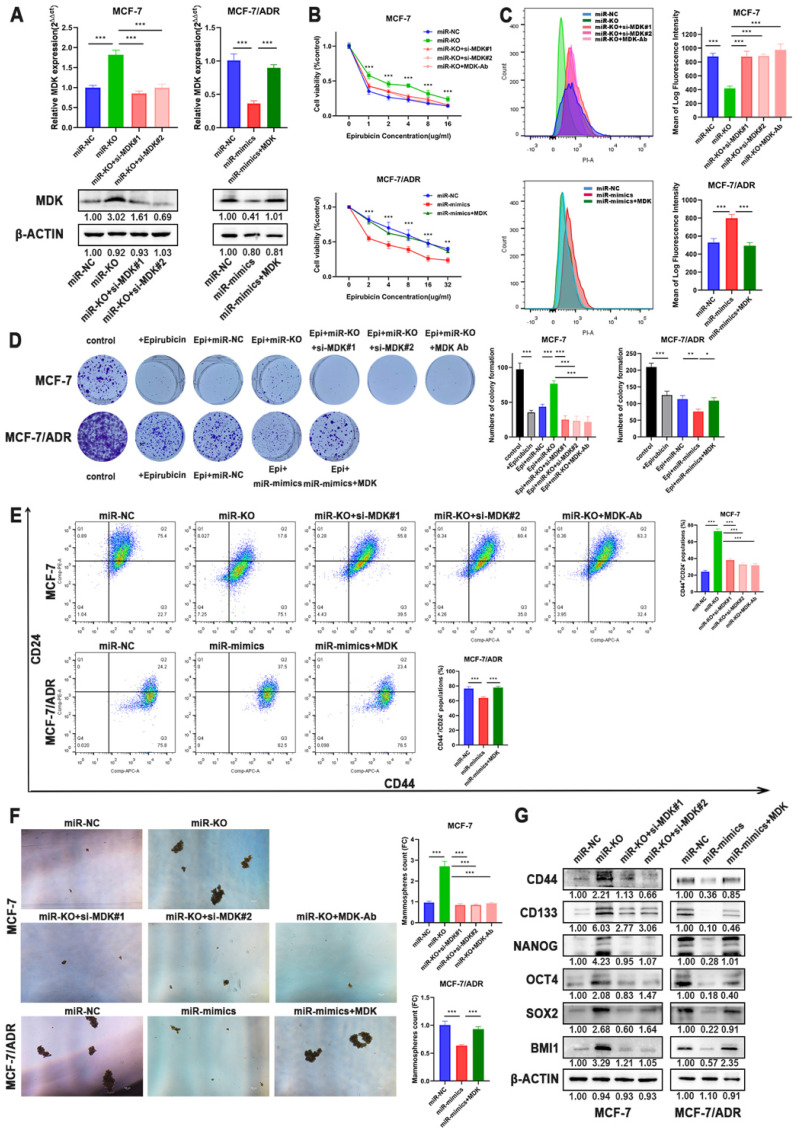
** Overexpression of MDK accounted for miR-1275 function in BC. A.** The mRNA and protein expression of MDK was measured by RT-qPCR and western blot. **B-D.** Drug resistance of MCF-7 and MCF-7/ADR transfected with miR-NC, miR-mimics, miR-knocked-out, miR-mimics and MDK plasmid, miR-knocked-out and MDK siRNAs or miR-knocked-out and MDK antibody were determined by CCK-8 assays (B.), flow cytometry analysis (C.), and colony formation experiments (D.). E-F CSC properties of MCF-7 and MCF-7/ADR transfected with miR-NC, miR-mimics, miR-knocked-out, miR-mimics and MDK plasmid, miR-knocked-out and MDK siRNAs or miR-knocked-out and MDK antibody were determined by flow cytometry analysis **(E.)** and mammosphere formation assay **(F.)**. **G.** CD44, CD133, NANOG, OCT4, SOX2 and BMI1 expression was measured by western blot in MCF-7 and MCF-7/ADR treated with miR-NC, miR-mimics, miR-knocked-out, miR-mimics and MDK plasmid or miR-knocked-out and MDK siRNAs. *P < 0.05, **P<0.01, ***P < 0.001. The data expressed as the mean ± SD. Scale bar, 100 µm.

To verify that MDK is the functional target of miR-1275, we performed rescue experiments in BC cells. RT-qPCR and Western blot analysis both confirmed the re-expression or knocking-down of MDK in BC cells (Fig. 5a, Supplementary Fig. 2a-b). Although miR-1275 overexpression suppressed chemoresistance in BC cells, CCK-8 assays confirmed that the chemotherapeutic sensitivity of epirubicin evidently abrogated by MDK plasmid in BC cells (Fig. 5b, Supplementary Fig. 2c). As for intracellular drug accumulation, overexpression of MDK markedly decreased the accumulation of epirubicin in cells induced by overexpression of miR-1275 (Fig. 5c, Supplementary Fig. 2d). Colony formation experiments also confirmed that inhibition of MDK reversed the increased cell proliferation during the treatment with epirubicin by silence of miR-1275 (Fig. 5d, Supplementary Fig. 2e). Flow cytometry revealed that MDK restoration improved the populations of CD44^+^/CD24^-^ in BC cells after the overexpression of miR-1275 (Fig. 5e, Supplementary Fig. 2f). Moreover, spheroids formation ability damaged by miR-1275 were ameliorated by MDK restitution (Fig. 5f, Supplementary Fig. 2g). The expression levels of the stem cell factors also changed under the effect of MDK, as MDK plasmid increased CD44, CD133, NANOG, OCT4, SOX2 and BMI1 expression, and vice versa (Fig. 5g, Supplementary Fig. 2b). In general, these results reflected that MDK was the functional target of miR-1275 and that miR-1275 suppressed the aggressive characteristics of BC cells by downregulating MDK.

**Figure 6:**
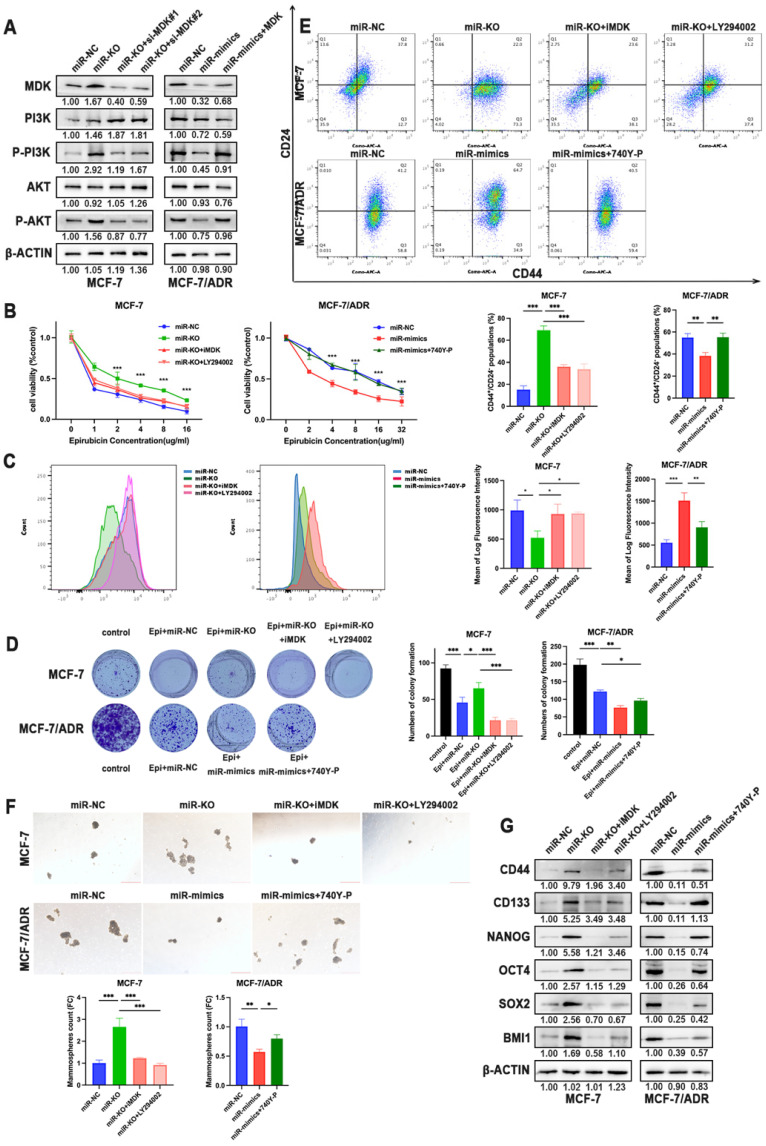
** The reduction of miR-1275 promotes BC cells chemoresistance via MDK/AKT axis. A.** MDK, PI3K, P-PI3K, AKT and P-AKT expression was measured by western blot in MCF-7 and MCF-7/ADR. **B-D.** Drug resistance of MCF-7 and MCF-7/ADR transfected with miR-NC, miR-mimics, miR-knocked-out, miR-mimics and 740Y-P, miR-knocked-out and iMDK or miR-knocked-out and LY294002 were determined by CCK-8 assays (B.), flow cytometry analysis (C.), and colony formation experiments (D.). **E-F.** CSC properties of MCF-7 and MCF-7/ADR transfected with miR-NC, miR-mimics, miR-knocked-out, miR-mimics and 740Y-P, miR-knocked-out and iMDK or miR-knocked-out and LY294002 were determined by flow cytometry analysis (E.) and mammosphere formation assay (F). **G.** CD44, CD133, NANOG, OCT4, SOX2 and BMI1 expression was measured by western blot in MCF-7 and MCF-7/ADR treated with miR-NC, miR-mimics, miR-knocked-out, miR-mimics and 740Y-P, miR-knocked-out and iMDK or miR-knocked-out and LY294002. *P < 0.05, **P<0.01, ***P < 0.001. The data expressed as the mean ± SD. Scale bar, 200 µm.

MDK has been verified to activate the Akt signaling pathway to provide cellular protection signal to doxorubicin. Therefore, we assumed PI3K-Akt signaling pathway as activated pathways related to chemoresistance. The silence of miR-1275 notably elevated the phosphorylation levels of PI3K and Akt proteins but not the expression of PI3K and Akt in MCF-7 cells, which could be rescued by MDK knockdown. On the opposite, MCF-7/ADR, SUM-1315 and MDA-MB-231 cells showed decreased activation of PI3K and Akt signaling upon miR-1275 overexpression, while the effect promoted by miR-1275 was released by MDK overexpression in the co-transfected group (Fig. 6a, Supplementary Fig. 2b). To further examine whether miR-1275 inhibited chemoresistance in BC cells by inhibiting PI3K/Akt signaling axis, miR-1275 knock-out cells were treated with MDK inhibitor iMDK (MedChemExpress) or PI3K/Akt inhibitor LY294002 (Beyotime). We observed that LY294002 treatment alleviated the inhibitory effects of miR-1275 on chemoresistance in MCF-7 cell lines (Fig. 6b-d). In parallel to decreased phosphorylation levels of PI3K and Akt, iMDK and LY294002 greatly reduced CSC percentage, prevented tumor sphere-forming capacity and reduced stemness-related protein expression in MCF-7(Fig. 6e-g). On the other hand, the PI3K/Akt signaling activator 740Y-P (MedChemExpress) displayed the opposite effects in BC cells with miR-1275 overexpression (Fig. 6b-g, Supplementary Fig. 2b-g). Collectively, the above-mentioned results confirmed that the miR-1275/MDK axis regulated PI3K-Akt signaling pathway, thereby facilitating chemoresistance in BC cells.

**Figure 7:**
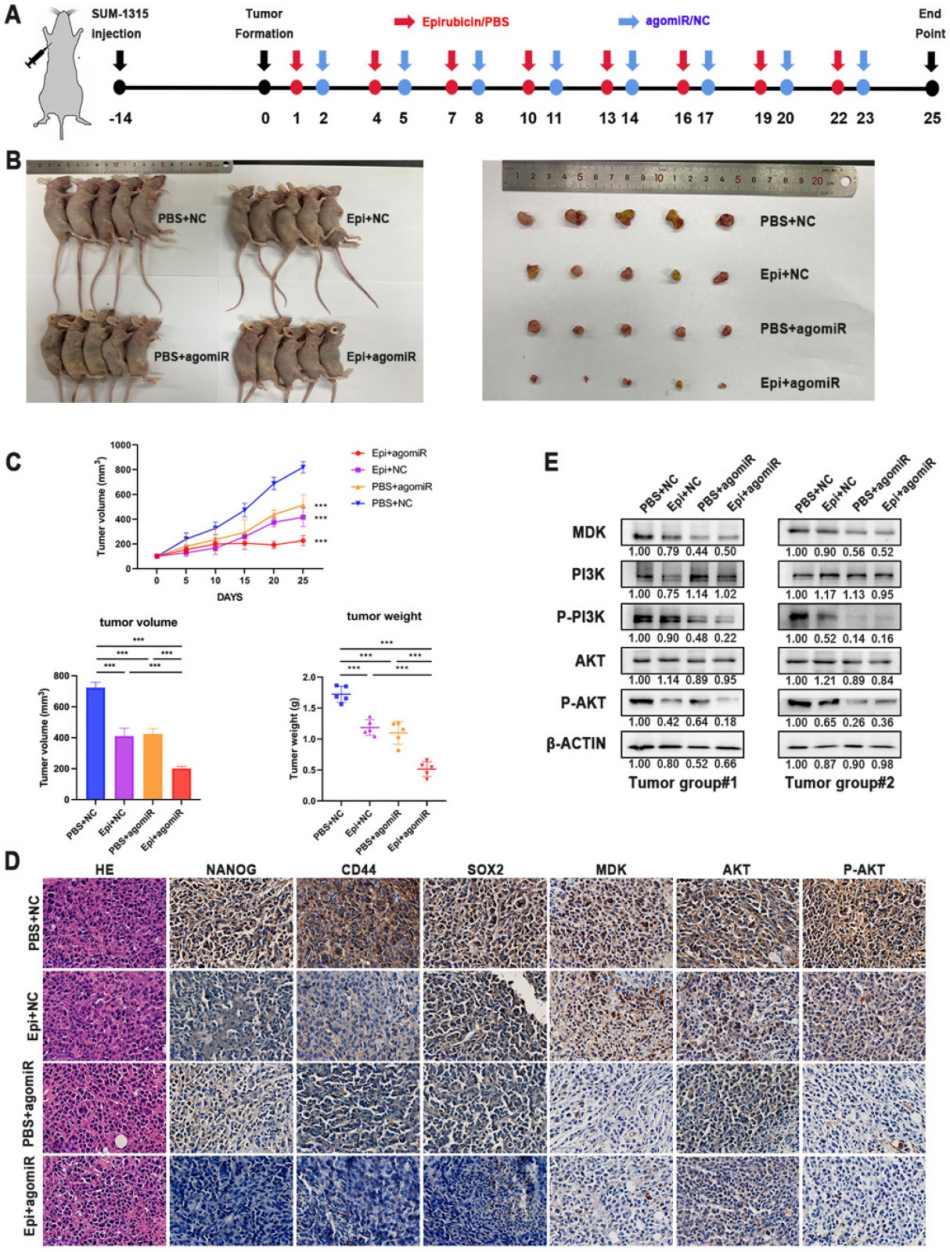
** Overexpression of miR-1275 reduced chemoresistance *in in vivo*. A.** *In in vivo* experimental design. **B.** Representative images of mice and tumors at the end point after subcutaneous transplantation when mice were euthanized. **C.** Tumor growth curves, tumor volume and tumor weight of the respective groups. **D.** IHC analysis of the expression levels of NANOG, CD44, SOX2, MDK, AKT and P-AKT in the respective groups. The positive staining was indicated by a brown color. **E.** MDK, PI3K, P-PI3K, AKT and P-AKT expression was measured by western blot in different tumors. ***p < 0.001. Scale bar, 50 µm.

To determine whether miR-1275 can affect the sensitivity of tumors to epirubicin *in in vivo*, SUM-1315 cells were used to establish subcutaneous xenografts in female BALB/c nude mice. When tumors reached an average of 100 mm^3^ (about 14 days), mice were randomly divided into four groups (n = 5/group). MiR-1275 mimic or control agomiR was released by intertumoral injection, and mice were injected intraperitoneally with epirubicin or PBS at the same time. The experimental schematic diagram is shown (Fig. 7a). After 25 days, all tumors weight were measured, and the results showed the tumor volumes and weight were dramatically decreased in the miR-1275 mimic agomiR plus epirubicin mice group (Fig. 7b-c). The tumor growth curves were also shown (Fig. 7c). The expression level of NANOG, CD44, SOX2 and MDK were detected by IHC (Fig. 7d). These results showed that miR-1275 reduces epirubicin resistance and CSC populations in BC through MDK *in in vivo*. To verify the regulation of miR-1275 on the PI3K/Akt pathway *in in vivo*, western blot and immunohistochemistry were used to determine the activity of the PI3K/Akt signaling pathway. Results of western blotting assay and IHC demonstrated that the level of PI3K/Akt signaling pathway was decreased in mice injected with miR-1275 mimic agomiR plus epirubicin (Fig. 7d-e). In addition, to confirm whether the effect of miR-1275 on chemoresistance *in in vivo* was in a dose-dependent way, we treated mice with different dose of miR-1275 mimic agomiR (1 mg/kg, 1.5 mg/kg, 2 ml/kg, 2.5 mg/kg, 3 mg/kg, respectively) (n=3/group). The experimental schematic diagram is the same as the previous (Supplementary Fig. 3a). The tumor weight and volumes were shown (Supplementary Fig. 3b-d). The results showed that along with the increase of miR-1275 mimic agomiR concentration, the tumor size decreased; however, there was no evidence for a statistically significant difference in the change of tumor size and weight when the concentration was over 2 mg/kg. This indicated that the influence of miR-1275 in chemoresistance is not dose-dependent *in in vivo*.

## Discussion

Chemotherapy is one of the main treatment strategies for BC. However, drug resistance remains a major problem leading to treatment failure or tumor recurrence. Therefore, it is imperative to identify drug resistance in the first place and exploit effective therapeutic methods to overcome chemotherapy resistance 15. Liquid biopsy is the sampling and analysis of blood or other bodily fluids to assess circulating tumor cells, nucleic acids, proteins and metabolites 6, 7, 15. The preliminary studies of our group showed that circulating miRNAs could be applied in the screening of BC patients, as we identified four plasma miRNAs and four serum miRNAs from the miR-106a-363 cluster, which could be used as new noninvasive biomarkers for BC detection 16. Continuous liquid biopsies can indicate the dynamic evolution of tumor, reflect heterogeneity, assess the variance of different metastatic lesions, observe the effect of treatment on tumor cells and monitor clonal evolution, which would promote understanding the mechanisms of chemoresistance and provide precise and individualized treatment plans in refractory tumors 17-22. We have explored the use of circulating miRNAs as prognostic markers for NCT, and identified miR-1275 was significantly associated to the response to NCT as well as the correlation to OS in LABC patients. Our results revealed that miR-1275 was significantly downregulated in plasma from patients resistant to chemotherapy with LABC and in chemoresistant breast cancer cell lines, while patients with low levels of miR-1275 show poor overall survival. These findings suggested that miR-1275 could act as a predictor of drug response in BC patients and a biomarker for clinical precision medicine.

Chemotherapeutic resistance can be caused by a variety of factors including changes in the biological characteristics of tumor cells, specifically, such as modulation of the status of breast cancer stem cells, fortified drug efflux relying on ATP-dependent pumps, enhanced repair of DNA damage, alterations of intracellular drug targets and impaired regulation of multiple signaling pathways 23-26. Cancer stem cells, characterized as CD44^+^/CD24^-/low^ expression and high ALDH activity, possess tumor initiation capacity, unlimited self-renewal competence and the ability of differentiating into heterogeneous cells 27. During chemotherapy, CSCs are in a dormant state and not affected by chemotherapy drugs, but eventually, the reactivation of CSCs can cause the cancer relapse 28. Because of these characteristics, CSCs promote tumor metastasis and chemotherapy resistance, and ultimately leading to poor prognosis of patients 29. In the current study, we detected the CD44^+^/CD24^-^ subpopulation cells from breast cancer cells, and confirmed that miR-1275 plays a pivotal role in decreasing chemoresistance by reducing the properties of CSCs.

Several studies have identified that miR-1275 participate in and regulates the genesis and progression of tumors 30-34. Xie et.al reported that in glioblastoma miR-1275 was involved in the modulation of a series of genes related to tumor progression, CSCs maintenance, cell maturation and differentiation, which provided a new therapeutic target for differentiation induction therapy 30. MiR-1275 inhibited the proliferation, invasion and migration of glioma cells, while promoted apoptosis via activating p53 signaling pathway 31. However controversially, Jiang et.al also found that miR-1275 played a carcinogenic role in the tumorigenesis, recurrence, and metastasis of lung adenocarcinoma 32. These paradoxical results suggested that the dominant function of miR-1275 should be related to specific cell types and therapies. In our study, we verified that miR-1275 had effects on BC chemotherapy resistance, as overexpression of miR-1275 conferred chemoresistance to BC cells *in in vitro* and *in in vivo*. Ectopic miR-1275 expression suppressed the sphere formation ability of breast cancer cells, whereas miR-1275 downregulation increased self-renewal and chemotherapy resistance.

To explore the latent mechanism of miR-1275 reduction leading to the induction of chemoresistance in BC via the increase of CSC proportion, the downstream of miR-1275 was investigated with both mechanical and functional experiments. The results indicated that miRNA-1275 could directly bind to the 3` UTR of MDK and overexpression of MDK could eliminate the role of miRNA-1275 in BC. AS a heparin-binding growth factor, MDK is abnormally high expressed in a variety of human malignancies and serves as an intermediary agent for the acquiring critical features of cancer, such as proliferation, metastasis, chemoresistance, migration, and angiogenesis 35-38. Some studies have shown that MDK can be served to monitor the response to tumor treatment, while the secretion and overexpression of MDK in chemoresistant cells can protect themselves from cannabinoid and doxorubicin treatment 39-42. Our findings uncovered that miR-1275-reduced chemoresistance was induced by the increase of MDK-mediated population of CSCs and PI3K/AKT phosphorylation and thus restored sensitivity of cells to epirubicin. Hence, we found that miR-1275 acted as a tumor suppressor by targeting MDK to reduce the properties of CSCs by inactivation of PI3K/AKT signaling pathway.

In conclusion, we have identified that the high expression level of miR-1275 in plasma predicted better response to NCT (Fig. 7a). The reduction of miR-1275 promotes BC cells chemoresistance by increasing CSC properties via targeting MDK/AKT axis (Fig. 7b). These results may uncover mechanisms of chemoresistance in BC, and be regarded as new prognostic biomarker and therapeutic targets of breast cancer patients.

## Materials and Methods

### Clinical samples

30 patients diagnosed with LABC from the First Affiliated Hospital of Nanjing Medical University were enrolled in the study. The standard set and the collection of plasma were performed as reported previously 8. To evaluate the response of NCT, Miller-Payne system grades were used, which were obtained from postoperative and preoperative biopsy pathology reports. Patients with miller-Payne grade 1-2 were categorized into a low response group (Low-R), while patients with miller-Payne grade 3-5 were classified as a high response group (High-R).

### Access and analysis of public data

The relevance between mR-1275 expression level and the overall survival rate of patients with LABC was analyzed with Kaplan-Meier Plotter (https://kmplot.com/analysis/). Auto select best cutoff was chose. The expression level of miR-1275 in BC cell lines was acquired from the Cancer Cell Line Encyclopedia (CCLE) database (http://www.broadinstitute.org/ccle). The IC50 values of epirubicin in corresponding cell lines were found from the Genomics of Drug Sensitivity in Cancer (GDSC) database. All transcripts were normalized by log2 transformation. The potential down streams of miR-1275 were identified in TargetScan (https://www.targetscan.org/), miRWalk (http://mirwalk.umm.uni-heidelberg.de), miRDB (http://mirdb.org), and DIANA (http://www.dianalab.gr). Possible binding sites between miR-1275 and 3'-UTR of MDK were obtained from TargetScan.

### Cell lines and culture condition

SUM-1315 was kindly provided by Stephen Ethier (University of Michigan, Ann Arbor, MI, USA). MDA-MB-231 and MCF-7 were purchased from the American Tissue Culture Collection (ATCC). All cell lines were cultured in DMEM medium (HyClone, Logan, UT, USA) supplied with 10% fetal bovine serum (Gibco, Detriot, MI, USA) and 1% penicillin-streptomycin (HyClone, Logan, UT, USA) at 37 °C with 5% CO2. The epirubicin resistant MCF-7/ADR cell line was established as described previously. Briefly, MCF-7 cells were first cultured with low-dose epirubicin for 1 month. The epirubicin concentration was then increased gradually, until the MCF-7/ADR resistant strain was obtained.

### Lentivirus and plasmid transfection

The negative control (NC), mimics lentiviruses (mimics) for miR-1275 were constructed by GenePharma (Shanghai, China) to alter the expression level in BC cell lines. Lentivirus transfection was performed on the basis of respective MOI. Puromycin (Sigma, USA) were used to select stable cell lines. Plasmid and siRNAs of MDK was constructed by GenePharma (Shanghai, China). Cells were seeded into 6-well plates one day before transfection, and transfections were performed with Lipofectamine 3000 (In*in vitro*gen, USA).

### CRISPR/Cas9 Experiments

Two sgRNAs fragments: 5'-TAAGGACTCCTCTGTGAGAA-3' and 5'-ACTCCGCAGCCACCGCATGG-3', were cloned into pCas-Puro-U6-KO vectors, respectively (Corues Biotechnology, Nanjing, China). Then, the plasmids were co-transfected into MCF-7 cells for 48h, and treated with 4 µg/ml puromycin for another 48h. Afterwards, the surviving cells were seeded into 96 well at a density of one cell per well for culturing about 7-10 days. Individual clones constructed with knockout of miR-1275 were expanded and screened for miR-1275 depletion by Sanger sequencing.

### CCK8 assay

After transfection, MCF-7/ADR, MCF-7, MDA-MB-231 and SUM-1315 cells were seeded into 96-well plates at a density of 1× 10^4^ cells/well. After 24 h, the cells were treated with Epirubicin hydrochloride (MedChemExpress, USA) for 24 h. Next, 10 µl of CCK-8 reagent (Beyotime, Shanghai, China) were added to cells one hour before the measurement of absorbance of 450 nm on a spectrophotometer.

### Colony formation assay

After transfection, MCF-7/ADR, MCF-7, MDA-MB-231 and SUM-1315 cells (2 × 10^3^ cells/well) in the logarithmic growth phase were resuspended and seeded into a 6-well plate. For the drug treatments, 1 μg/ml epirubicin was added to MCF-7, MDA-MB-231 and SUM-1315 cells and 2 μg/ml epirubicin was added to MCF-7/ADR cells for 1 week. Subsequently, the culture medium was replaced with normal medium. Two weeks later, the cells were washed, fixed and stained with 0.1% crystal violet (Beyotime, China) for 30 min.

### Flow cytometry analysis

For epirubicin accumulation assay, the MCF-7/ADR cells were exposed to 2 μg/ml epirubicin, while 1 μg/ml epirubicin was added to MCF-7, MDA-MB-231 and SUM-1315 cells and then after 24h cells were washed with PBS. Then the intracellular epirubicin level was detected by flow cytometry.

Flow cytometry was used to analyze the expression levels of CD44 (Cluster of differentiation 44) and CD24 (Cluster of differentiation 24). Briefly, cells were isolated with Trypsin Solution without EDTA (Beyotime, Shanghai, China) and resuspended. After incubating with anti-human CD44-APC, anti-human CD24-PE (eBioscience, USA) for 30 minutes at room temperature, cells were washed with PBS and evaluated by flow cytometry (BD Biosciences, USA).

### Mammosphere formation assay

After transfection, cells were seeded into ultra-low attachment 6-well plates (Corning, USA) at a density of 5,000 cells/well in DMEM/F12 medium with additional B27 (In*in vitro*gen), 20 ng/ml EGF (In*in vitro*gen), and 20 ng/ml bFGF (BD Biosciences). After 1-2 weeks, the number of mammospheres whose diameters were larger than 60 μm was counted under the microscope.

### RNA extraction and quantitative real time-polymerase chain reaction (RT-qPCR)

Total RNA from tissues and cells was extracted using Trizol reagent (Takara, Japan) following the manufacturer's protocol. Total RNA from plasma and cell supernatants were extracted by the mirVana PARIS Kit (In*in vitro*gen, Lithuania). For miRNA expression analysis, cDNA was specifically synthesized and miRNA was detected with Bulge-LoopTM miRNA RT-qPCR (Ribobio, Guangzhou, China). Relative expression level of miR-1275 was normalized to U6. For mRNA expression analysis, first strand cDNA was synthesized by PrimeScript™ RT Master Mix (Takara, Japan). The RT-qPCR reactions were performed with TB Green® Premix Ex Taq™ II (Takara, Japan) on the Roche LightCycler® 480 System (Roche, Switzerland).

### Western blot

Western blot (WB) was performed as reported previously 43. The primary antibodies included anti-CD44, anti-CD133, anti-NANOG, anti-OCT4, anti-SOX2, anti-BMI1, anti-MDK, anti-PI3K-p85, anti-p-PI3K-p85, anti-AKT, anti-p-AKT and anti-ACTIN (diluted 1:1000, Cell Signaling Technology, USA). ACTIN was used as an internal control.

### Biotinylated miRNA pull-down

After transfecting the biotinylated miR-NC or mimics (Ribobio, Guangzhou, China) into MCF-7/ADR and MDA-MB-231 cells for 24h, the cells were digested and crosslinked to prepare lysate. In accordance with the protocol, the crude lysate was incubated with Streptavidin C1 (In*in vitro*gen, USA), and the biotin-miRNA-mRNA were isolated. Trizol reagent was used to extract RNA from the remaining beads.

### Luciferase reporter assay

Wild-type MDK plasmid (MDK-WT) and mutant MDK plasmid (MDK-MUT) were purchased from Genebay (Nanjing, China). After transfecting appropriate plasmid to the cells for 48h, luciferase assays were performed with the Dual-luciferase reporter assay system (GeneCopoeia, USA). Luciferase activity/ Renilla Luciferase activity was defined as transcriptional activity.

### Collection of Conditioned Media (CM)

The stably transfected cells were seeded in serum-free and antibiotic free medium at a density of 1×10^6^ cells per well in a 6-well plate. CM were collected 24h later and centrifuged to remove any residual cells, followed by filtering with a 0.22 µm pore size syringe filter. After collecting CM, the number of cells in the dish was counted and normalized according to the volume of CM used in each experiment.

### MDK ELISA Assay

The collected media was cryopreserved at -80 °C, and thawed on ice before analysis. MDK concentrations in the culture media of the cells transfected with miR-1275 were measured with an enzyme-linked immunosorbent assay (ELISA) kit for human Midkine (RayBio Inc., Norcross, WA, USA), and determined according to the manufacturer's protocol.

### Animal models

All animal experiments were approved by the Animal Center of Nanjing Medical University. All BALB/c nude mice (Female, 4-6 weeks old) were purchased from Vital River Laboratory Animal Technology (Beijing, China). SUM1315 cells (1 × 10^7^ cells/100 μl per nude mice) were injected into the axillary region of the nude mice to establish subcutaneous xenograft tumor model. When the average size of subcutaneous xenografts in each group reached 100 mm^3^ (nearly 2 weeks later), nude mice in the treatment group were intraperitoneally injected with epirubicin (2 mg/kg) and/or agomiR-1275 (20 mg/kg; Ribobio, Guangzhou, China) every 3 days. For the negative control, we use PBS and agoNC (20 mg/kg; Ribobio, Guangzhou, China) respectively. The diameter of xenograft tumor was measured every 5 days, and the volume was calculated following the formula: volume = 0.5 × width^2^ × length. All nude mice were humanly sacrificed after 8 period of treatment.

### Immunohistochemical analyses (IHC)

After slicing the formalin-fixed paraffin blocks into four-micrometer-thick sections, they were dried, dewaxed, and rehydrated in accordance with the protocol. After antigen retrieval, anti-MDK, anti-CD44, anti-SOX2, anti-NANOG, anti-AKT and anti-p-AKT were incubated at room temperature for 1 h. Standard avidin‑biotin‑peroxidase techniques were used for detections. Immunohistochemical specimens were evaluated by two pathologists.

### Statistical analysis

SPSS software (Version 25.0) and GraphPad Prism (Version 8.0) were used for statistical analysis, which was presented as mean ± standard deviation (SD). All data were analyzed by two-tailed Student's t-test. All experiment were repeated at least three times, and p < 0.05 was considered statistically significant.

## Supplementary Material

Supplementary figures.

## Figures and Tables

**Figure 8:**
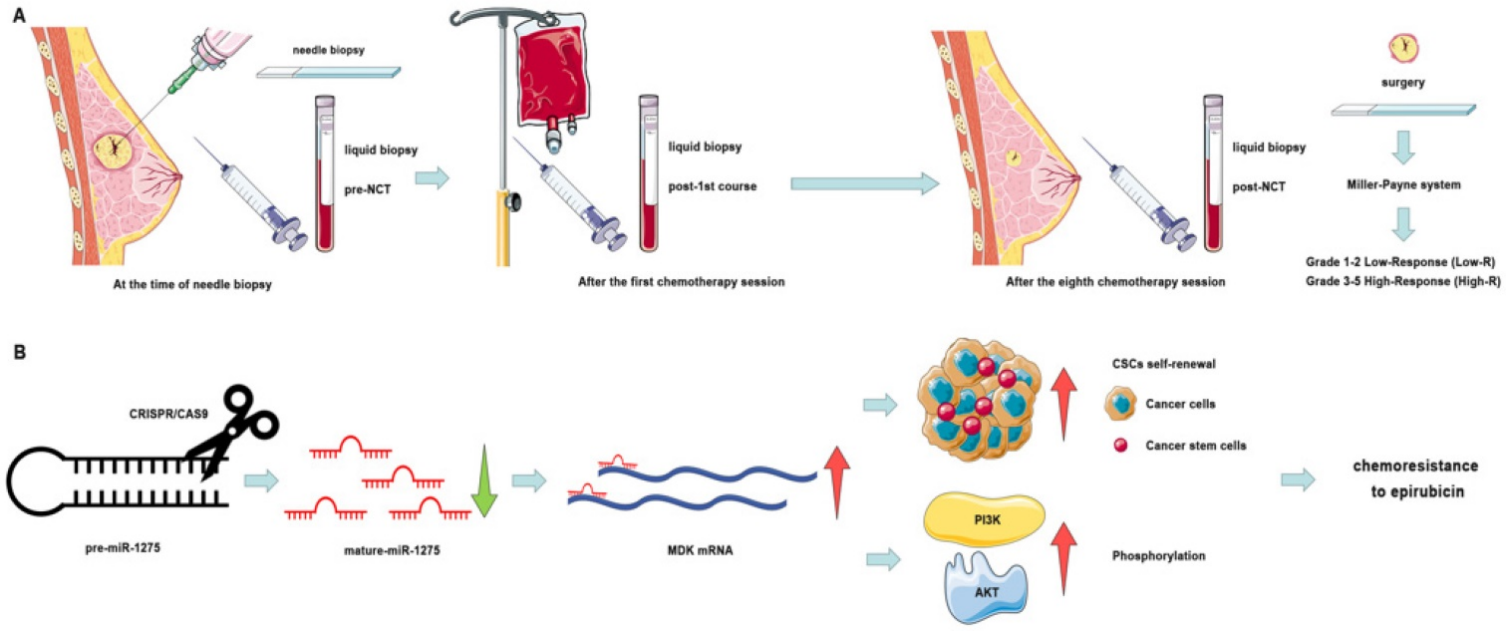
** Schematic diagram of the study. A.** Schematic diagram of clinical study. **B.** Schematic diagram of miR-1275 in BC and its mechanism.

**Table 1:** Clinical characteristics of patients with locally advanced breast cancer receiving neoadjuvant chemotherapy in the study

Factors	Low-R (No.)	High-R (No.)	P value
**Age (y)**			
<50	9	6	0.4661
≥50	6	9	
**HER-2 status**			
Negative	11	8	0.4497
Positive	4	7	
**Molecular subtype**			
Hormone+ HER-2-/+	12	7	0.09
TNBC	3	5	
Hormone- HER-2+	0	3	
**Lymph node**			
≤2	10	8	0.7104
>2	5	7	
