# Supplementary material for: miR-1275 targets MDK/AKT signaling to inhibit breast cancer chemoresistance by lessening the properties of cancer stem cells
Source: Int J Biol Sci. 2023 Jan 1;19(1):89–103. doi: 10.7150/ijbs.74227 (PMC9760432; doi:10.7150/ijbs.74227)
Supplement: Supplementary file 1 — Supplementary figures. [file ijbsv19p0089s1.pdf]

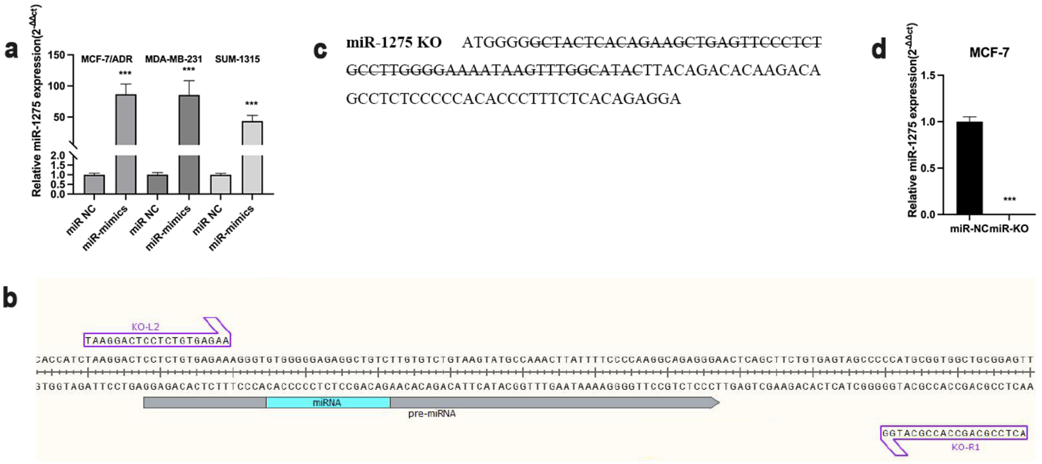

Supplementary Fig. 1 the expression levels of miR-1275 in breast cancer cells were verified after transfection. A. The expression levels of miR-1275 in MCF-7/ADR, MDA-MB-231 and SUM-1315 transfected with mimics lentivirus were tested by RT-qPCR. B. The design of CRISPR/Cas9 sgRNA to construct miR-1275 knock-out MCF-7 cells. C. The sequence character of miR-1275 was confirmed with Sanger sequencing in MCF-7 cells. D. The expression level of miR-1275 in gene-edited MCF-7 cells was tested by RT-qPCR. *$p < 0.05$, **$p<0.01$, ***$p < 0.001$. The data expressed as the mean ± SD.

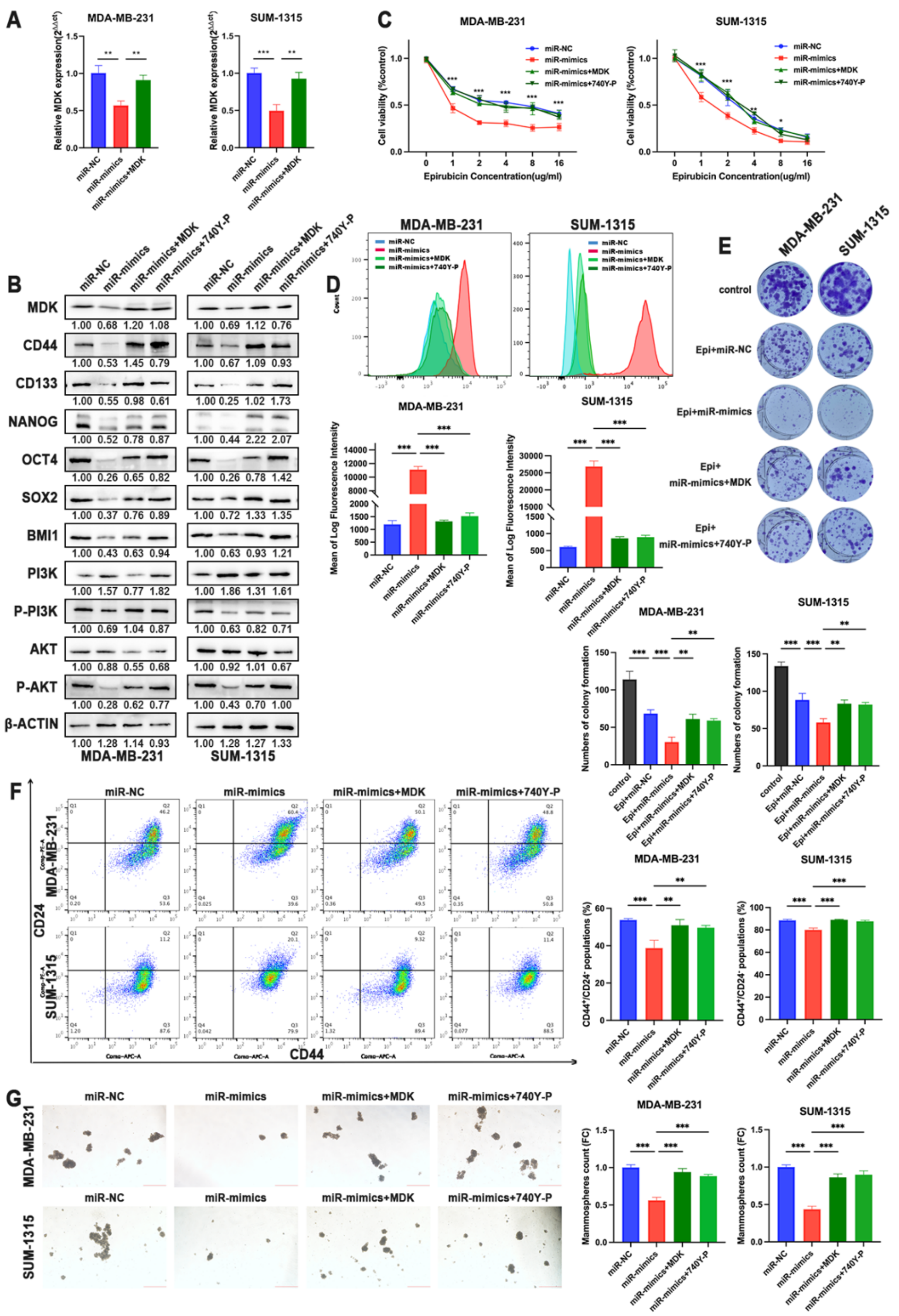

Supplementary Fig. 2 The reduction of miR-1275 promotes BC cells chemoresistance via MDK/AKT axis. A. The mRNA expression of MDK was measured by RT-qPCR in MDA-MB-231 and SUM-1315. B. MDK, CD44, CD133, NANOG, OCT4, SOX2, BMI1, PI3K, P-PI3K, AKT and P-AKT expression was measured by western blot in MDA-MB-231 and SUM-1315 treated with miR-NC, miR-mimics, miR-mimics and MDK plasmid or miR-mimics and 740Y-P. C-E. Drug resistance of MDA-MB-231 and SUM-1315 transfected with miR-NC, miR-mimics, miR-mimics and MDK plasmid or miR-mimics and 740Y-P were determined by CCK-8 assays (C.), flow cytometry analysis (D.), and colony formation experiments (E.). F-G. CSC properties of MDA-MB-231 and SUM-1315 transfected with miR-NC, miR-mimics, miR-mimics and MDK plasmid or miR-mimics and 740Y-P were determined by flow cytometry analysis (F.) and mammosphere formation assay (G). *P < 0.05, **P<0.01, ***P < 0.001. The

data expressed as the mean ± SD. Scale bar, 200 μm.

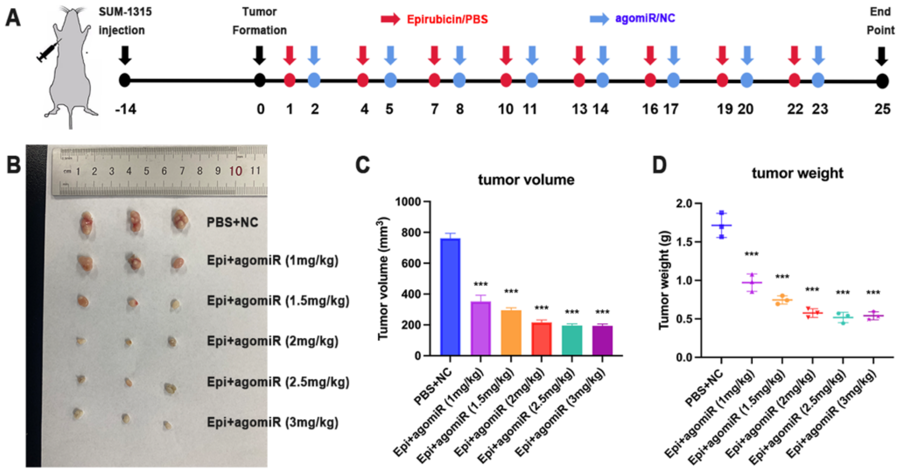

Supplementary Fig. 3 Different dose of agomiR-1275 reduced chemoresistance in vivo. A. In vivo experimental design. B. Representative images of mice and tumors at the end point after subcutaneous transplantation when mice were euthanized. C. Tumor volume of the respective groups. D. Tumor weight of the respective groups. *P < 0.05, **P<0.01, ***P < 0.001. The data expressed as the mean ± SD.
